# STIP overexpression confers oncogenic potential to human non‐small cell lung cancer cells by regulating cell cycle and apoptosis

**DOI:** 10.1111/jcmm.12670

**Published:** 2015-09-10

**Authors:** Yani Tang, Guobei Yan, Xin Song, Kuangpei Wu, Zhen Li, Chao Yang, Tanggang Deng, Yang Sun, Xiaoxiao Hu, Cai Yang, Huarong Bai, Hui Li, Weihong Tan, Mao Ye, Jing Liu

**Affiliations:** ^1^ Molecular Science and Biomedicine LaboratoryState Key Laboratory for Chemo/Biosensing and ChemometricsCollege of BiologyCollege of Chemistry and Chemical EngineeringCollaborative Innovation Center for Molecular Engineering for TheranosticsHunan UniversityChangshaHunanChina; ^2^ Cancer Biotherapy CenterTumour Hospital of Yunnan Province Affiliated with Kunming Medical UniversityKunmingYunnanChina; ^3^ School of Life SciencesState Key Laboratory of Medical GeneticsCentral South UniversityChangshaHunanChina

**Keywords:** lung cancer, cell cycle, apoptosis, caspase, proliferation

## Abstract

Sip1/tuftelin‐interacting protein (STIP), a multidomain nuclear protein, is a novel factor associated with the spliceosome, yet its role and molecular function in cancer remain unknown. In this study, we show, for the first time, that STIP is overexpressed in non‐small cell lung cancer (NSCLC) tissues compared to adjacent normal lung tissues. The depletion of endogenous STIP inhibited NSCLC cell proliferation *in vitro* and *in vivo*, caused cell cycle arrest and induced apoptosis. Cell cycle arrest at the G2/M phase was associated with the expression and activity of the cyclin B1‐CDK1 (cyclin‐dependent kinase 1) complex. We also provide evidence that STIP knockdown induced apoptosis by activating both caspase‐9 and caspase‐3 and by altering the Bcl‐2/Bax expression ratio. RNA sequencing data indicated that the MAPK mitogen‐activated protein kinases, Wnt, PI3K/AKT, and NF‐κB (nuclear factor kappa‐light‐chain‐enhancer of activated B cells) signalling pathways might be involved in STIP‐mediated tumour regulation. Collectively, these results suggest that STIP may be a novel potential diagnostic and therapeutic target for NSCLC.

## Introduction

Lung cancer is the most common type of cancer in the world and remains the leading cause of cancer‐related mortality 1, 2. The major types of lung cancer are classified histologically as non‐small cell lung cancer (NSCLC) and small cell lung cancer based on the tumour cell size 3. Non‐small cell lung cancer accounts for 80% of all lung cancer cases and is the most prevalent type of lung cancer 4. The majority of NSCLC patients are diagnosed at an advanced stage 5. Despite advances in surgery, radiotherapy and chemotherapy, the prognosis for patients with NSCLC remains poor, with a 5‐year survival rate of only 15% 6. Therefore, it is imperative to identify new molecular targets that play a role in the pathogenesis of NSCLC to design novel preventive/therapeutic strategies to control this malignancy.

Sip1/tuftelin‐interacting protein (STIP, also referred to as TFIP11) is a member of a unique class of multidomain proteins that share a G‐patch, a coiled‐coil domain and several short tryptophan–tryptophan repeats from the N‐ to C‐terminal regions. Sip1/tuftelin‐interacting proteins can self‐aggregate into large rod‐shaped polymers termed stiposomes in the nucleoplasm 7. In *Caenorhabditis elegans*, the knockdown of STIP *via* RNA interference causes developmental arrest of early embryos at the 16‐cell stage followed by 100% lethality. This lethal phenotype can be readily rescued with either human or *Drosophila* STIP, highlighting its conserved essential biological function across metazoans from worms to flies to humans 7.

Proteomic studies identified STIP as a nuclear phosphoprotein in HeLa cells 8 and as a spliceosome‐associated factor that mediates the release of the lariat intron during late‐stage splicing *via* its interaction with DHX14/PRP43 9, 10, 11, 12. The spliceosome, a large complex that includes small nuclear ribonucleoproteins (snRNPs) and non‐snRNP‐associated proteins, processes pre‐mRNA by excising intronic nucleic acids, thereby producing mRNA that is then translated into protein by ribosomes 13. Extensive studies have indicated that splicing events play an essential role in normal development and cell differentiation. The misregulation of splicing contributes to many aspects of cancer progression, including regulation of the cell cycle and apoptosis, cancer cell metabolism, angiogenesis and metastasis 14, 15. However, the biological roles and molecular functions of STIP in cancer remain unknown.

In this study, we first established the association between STIP expression and NSCLC and then investigated the functional role of STIP in tumourigenesis, cell cycle regulation and apoptosis induction in NSCLC cells. We also analysed the potential pathways involved in STIP‐mediated tumour regulation *via* RNA sequencing. Collectively, our results suggest that STIP might be a novel potential diagnostic marker and therapeutic target for NSCLC.

## Materials and methods

### Lung cancer tissue samples and cell lines

Fifty pairs of lung cancer and their corresponding adjacent normal tissues were obtained from lung cancer patients. The fresh specimens were snap‐frozen in liquid nitrogen and stored at −80°C until analysis. The human lung cancer cell lines A549 and H460 were maintained in RPMI‐1640 (Gibco BRL Co. Ltd., Grand Island, NY, USA) medium supplemented with 10% foetal bovine serum (Gibco) at 37°C under a humidified atmosphere containing 5% CO_2_.

### Western blot analysis

Whole cell lysates were prepared from lung cancer cells. Protein concentrations were determined by a BCA (bicinchoninic acid) protein assay kit (Pierce, Rockford, IL, USA). Standard Western blotting was done with a rabbit antibody against human TFIP11 (Bethyl Laboratories, Inc., Montgomery, TX, USA) or anti‐cyclinB1, anti‐ p‐cdc2 (Thr14/Tyr15), anti‐ p‐cdc2 (Thr161), anti‐Bax, anti‐Bcl‐2 and anti‐poly (ADP‐ribose) polymerase (PARP) antibodies (SantaCruz Biotechnology, Santa Cruz, CA, USA) or anti‐CDK1 and anti‐cdc25C antibodies (Sangon Biotechnology, Shanghai, China) and a secondary antibody (antirabbit IgG or antimouse IgG; SantaCruz Biotechnology). The same membranes were stripped and blotted with an anti‐GAPDH antibody (KangChen Bio‐tech Inc., Shanghai, China) and used as loading controls.

### Immunohistochemistry

Formalin‐fixed, paraffin‐embedded samples were sectioned at 5 μM. Sections were treated with antigen retrieval buffer. Specifically TFIP11 antibody was applied overnight at room temperature at a dilution of 1:100. Slides were incubated in secondary antibody. Immnostaining was carried out using standard techniques.

Levels of STIP expression in lung cancer tissues and corresponding normal lung tissue specimens from NSCLC patients were reviewed and scored under a light microscope by two independent pathologists (Song X and Li Z) who were not aware of the clinicopathological data. If there was a discrepancy, a consensus interpretation was reached under a two‐headed microscope. For STIP, cytoplasm and nuclear staining of ≥10% of the cancer cells was considered positive. If fewer than 10% of cytoplasm or nuclear was stained, the slides were scored as negative STIP expression. The STIP expression was quantified by a visual grading system (0–3) based on the intensity of cytoplasm and nuclear staining as follows: grade 0, no immunoreactivity; grade 1, weak immunoreactivity slightly stronger than background staining; grade 2, clear immunoreactivity in more than half of the cancer cells; grade 3, strong immunoreactivity as dark as nuclear counter stain in the majority of cancer cells.

### RNA interference

Pre‐designed STIP siRNA duplexes (sense sequence: 5′‐TGGGTTGGAAGTCGATGTT‐3′) and negative control siRNAs (5′‐TTCTCCGAACGTGTCACGTTTC‐3′) were purchased from GenePharma (Shanghai, China). A549 and H460 cells were transfected with STIP or control siRNA by Genmute transfection reagent (SignaGen, Gaithersburg, MD, USA) following the manufacturer's instruction. To stably knockdown endogenous STIP in some case, we used lentivirus packing shRNA expression vector (purchased from GenePharma) to infect A549 and H460 cells. Sip1/tuftelin‐interacting protein shRNA target sequences were 5′‐GTGGATCTTAGATAACATA‐3′. The control shRNA sequence was 5′‐TTCTCCGAACGTGTCACGTTTC‐3′.

### Cell proliferation assay

The effect of STIP knockdown on cell proliferation was determined by MTT (3‐(4,5‐Dimethylthiazol‐2‐yl)‐2,5‐Diphenyltetrazolium Bromide) assay. A549 and H460 cells were transfected with STIP shRNA or control shRNA, and the cells were seeded at a density of 5000 cells per well in 96‐well plates. At the indicated time‐points, the 3‐(4,5‐dimethylthiazol‐2‐yl)‐2,5‐diphenyltetrazolium bromide (Sigma‐Aldrich, St. Louis, MO, USA) solution was added to each well and incubated at 37°C for another 4 hrs. The supernatants were then aspirated carefully, and the formazan product was dissolved with 100 μl dimethyl sulfoxide. The absorbance was measured at a wavelength of 570 nm with a microplate reader (Bio‐Tek, Doraville, GA, USA).

### Colony formation assay

Briefly, 10,000 STIP shRNA‐ or control shRNA‐transfected cells were seeded in 6‐well plates. Cells were selected with 1.0 μg/μl puromycin and allowed to form colonies. After 10 days of culture, colonies were stained with 0.01% crystal violet, and the number of colonies was counted.

### Cell cycle analysis

Cells in 60‐mm culture plates were treated with STIP shRNA or control shRNA for 72 hrs. The cells were then harvested and washed in DPBS(Dulbecco's phosphate‐buffered saline), followed by fixation with ice‐cold 70% ethanol and stored at −20°C overnight. Next, the pellets were washed with cold DPBS, stained with propidium iodide (PI; 10 μg/ml), and incubated at 37°C for 30 min. in the dark. The stained cells were detected using flow cytometry (BD FACSVerse^™^, BD BioSciences, Franklin Lakes, NJ, USA).

### Morphological analysis of nuclei

Apoptotic morphological changes in the nuclear chromatin of cells were detected by Hoechst 33258 staining. A549 and H460 cells were seeded at a density of 100,000 cells in 35‐mm culture plates, allowed to recover overnight, then treated with STIP shRNA or control shRNA for 72 hrs. Following treatment, treated cells were fixed for 10 min. at room temperature. Hoechst 33258 was added to the cells, incubated for 5 min. at room temperature and washed with PBS twice. Then, images were taken using an inverted fluorescence microscope (Olympus, Dongjing, Japan).

### Detection of apoptosis

Activation of apoptosis signalling in A549 and H460 cells was assessed by Annexin V/PI double staining assay using FITC Annexin V Apoptosis Detection kits (Beyotime Institute of Biotechnology, Haimen, China). Floating cells were combined with cells detached by trypsinization, then subjected to centrifugation, washing in cold PBS and resuspension in Annexin V/PI binding buffer. The cells were then stained according to the manufacturer's instructions. The stained cells were analysed by flow cytometry (BD Biosciences).

### Caspase‐3, caspase‐8 and caspase‐9 activity assay

Briefly, cells were treated with STIP shRNA or control shRNA for 72 hrs and then lysed with lysis buffer on ice for 15 min. The lysates were centrifuged at 20,800g at 4°C for 15 min., the supernatants collected and protein concentration determined by BCA protein assay (Pierce). Cellular extracts (100 μg) were then incubated in a 96‐well plate with 20 ng Ac‐DEVD‐pNA (caspase‐3 activity), Ac‐IETD‐pNA (caspase‐8 activity) or Ac‐LEHD‐pNA (caspase‐9 activity) (Beyotime Institute of Biotechnology) at 37°C for 6 hrs. Caspase activity was measured by cleavage of the Ac‐DEVD‐pNA or Ac‐IEVD‐pNA or Ac‐LEHD‐pNA substrate to pNA, which was measured at 405 nm using a microplate reader (Bio‐Tek).

### Quantitative RT‐PCR

Total RNAs were isolated from A549 cells by using the Rnaprep Pure Kit (TIANGEN, Beijing, China) and converted to cDNAs using the ReverTra Ace qRCR RT Master Mix with gDNA Remover (Toyobo, Osaka, Japan). The amount of mRNA was measured by quantitative RT‐PCR with the KOD SYBR qPCR Mix (Toyobo, Osaka, Japan), according to the manufacturer's instructions, and GAPDH was used as an internal control. The primer sequences were as follows: GAPDH forward: 5′‐AAGGTGAAGGTCGGAGTCAA‐3′, reverse: 5′‐AATGAAGGGGTCATTGATGG‐3′; STIP forward: 5′‐CCAAATCTTTCATGGACTTCGGCAG‐3′, reverse: 5′‐GCTTCTTCCTCTGAGTCAACCACAG‐3′; GADD45A forward: 5′‐GCCTGTGAGTGAGTGCAGAA‐3′, reverse: 5′‐ATCTCTGTCGTCGTCCTCGT‐3′; GADD45B forward: 5′‐TCGGATTTTGCAATTTCTCC‐3′, reverse: 5′‐GGATGAGCGTGAAGTGGATT‐3′; NR4A1 forward: 5′‐TCTGCTCAGGCCTGGTGCTAC‐3′, reverse: 5′‐GGCACCAAGTCCTCCAGCTTG‐3′; NR4A3 forward: 5′‐CCTTGTCCGAGCTTTAACAG‐3′, reverse: 5′‐GGCACCAAGTCCTCCAGCTTG‐3′; NFATC4 forward: 5′‐TCTTCAGGACCTCTGCCCTA‐3′, reverse: 5′‐GCCACCATCTTGCCAGTAAT‐3′; EFNA1 forward: 5′‐AGGTGCGGGTTCTACATAGCA‐3′, reverse: 5′‐AGTCCAGGCAAGTGGGAAGA‐3′.

### *In vivo* tumour formation assay

To establish lung cancer xenografts in nude mice, a total of 5 × 10^6^ A549 cells in log phase stably transfected with either control or STIP targeting shRNA vectors were harvested, washed twice with DPBS, suspended in 100 μl DPBS and injected into the right flank site of each mouse (*n* = 6 for each group). All the mice were kept in pathogen‐free environments, and the xenografts were evaluated by calliper every 2 days for 1 month. Tumour volume was calculated according to the following formula: V = 0.5 (length × width^2^). All the mice were sacrificed at day 35.

### Statistical analysis

Statistical Package for Social Science (SPSS) version 19.0 for windows (SPSS Inc., Chicago, IL, USA) and GraphPad Prism 6 (GraphPad Software Inc., San Diego, CA, USA) were used to analyse the data. Student's *t*‐test was used to compare the data between every two groups respectively. For all statistical analysis, *P* value less than 0.05 was considered statistically significant.

## Results

### STIP was overexpressed in NSCLC tissues

**Figure 1:**
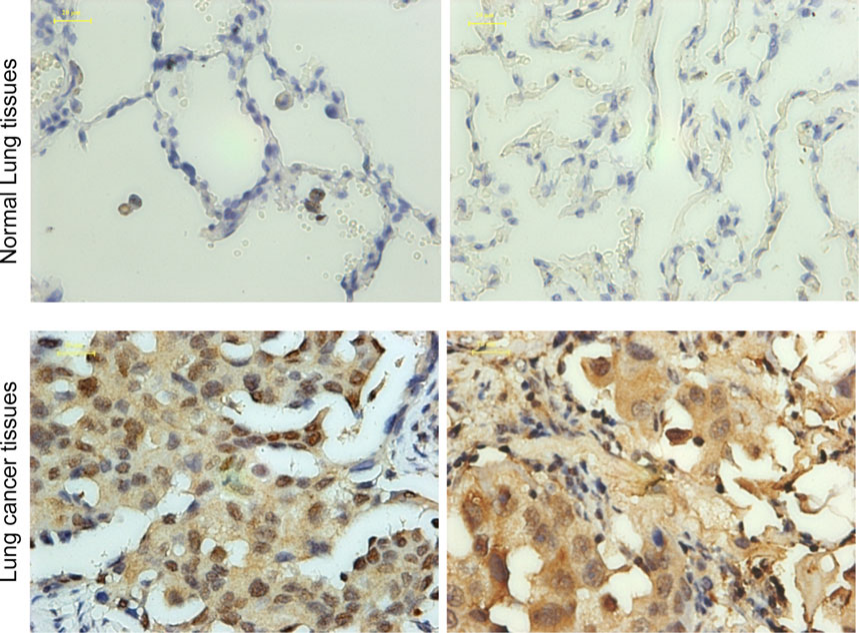
STIP protein expression in human lung cancer tissues and corresponding normal lung tissues. The typical IHC (Immunohistochemistry) results of STIP in human lung cancer tissues and corresponding normal lung tissues were shown. Two independent pathologists estimated the 100 specimens. If fewer than 10% of cytoplasm or nuclear was stained, the slides were scored as negative STIP expression.

To investigate the protein expression and intracellular localization of STIP, we performed immunohistochemical staining on 50 NSCLC tissues and 50 adjacent normal lung tissues using an antibody specific for STIP. We observed STIP immunoreactivity in both the cytoplasmic and nuclear compartments, but stronger immunohistochemical signal appear in nuclei (Fig. 1). Overall, 76% of NSCLC tissues were positive for STIP expression, and 24% were negative (Fig. 1 and Table 1). In contrast, 42% of normal lung tissues were positive for STIP expression, and 58% were negative. Analysis of the staining data revealed significantly higher positivity for STIP expression in NSCLC tissues than in adjacent normal lung tissues (*P* < 0.01) (Fig. 1 and Table 1). However, no association with prognoses was identified.

**Table 1:** Expression of STIP in lung cancer tissues and corresponding normal lung tissues of NSCLC patients

Characteristic	No. of samples	STIP		IHC score				
		No. of positive samples (%)	Statistical significance	No. of samples under different IHC score				Statistical significance
				0	1	2	3	
Lung cancer	50	38 (76.0)	*P* = 0.001	12	25	8	5	*P* = 0.000
Normal lung tissue	50	21 (42.0)		29	16	4	1	

### Down‐regulation of STIP‐inhibited lung cancer cell proliferation

**Figure 2:**
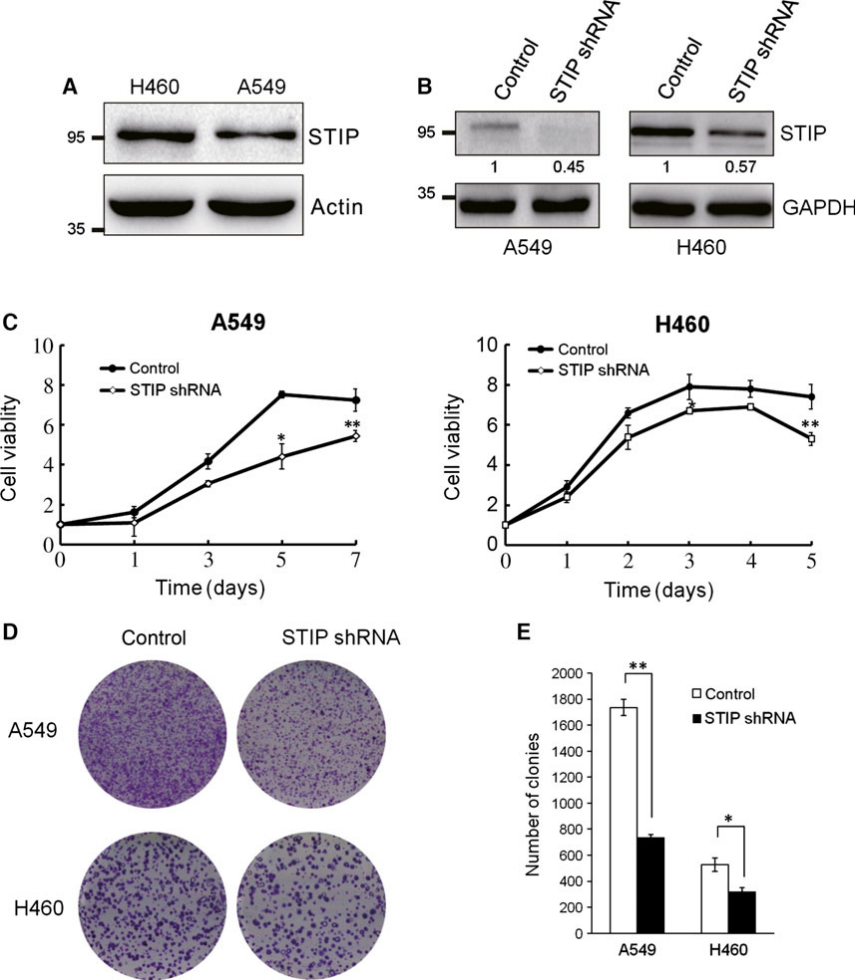
STIP suppressed cell growth. A549 cells and H460 cells were transfected with STIP or control shRNA. (**A**) The expression of endogenous STIP protein in A549 and H460 cells were examined by Western blot using indicated antibodies. Actin was used as an internal control. (**B**) A549 and H460 cells were infected with STIP shRNA, and the expression of STIP was examined by Western blot. GAPDH was used as an internal control. (**C**) Growth curve of the A549 and H460 cells. Cell viability was determined *via* the MTT assay at the indicated time‐points, *P < 0.05, **P < 0.01. (**D** and **E**) Colony formation assay of A549 cells and H460 cells. Cells were selected in the presence of 1 mg/ml puromycin for 10 days. Colonies were stained with 0.1% crystal violet and were subsequently photographed (**D**) and counted (**E**), *P < 0.05, **P < 0.01.

Because immunohistochemical analysis showed aberrant expression of STIP in NSCLC tissues, we suggested that STIP might be involved in the proliferation of lung cancer cells. To investigate this, endogenous STIP was depleted using STIP shRNA in A549 and H460 NSCLC cells. Compared with A549 cells, basal expression levels of STIP is higher in H460 cells (Fig. 2A). In spite of this, the expression of STIP was successfully knocked down using STIP shRNA in A549 and H460 cells (Fig. 2B). Subsequently, we assessed the effects of STIP knockdown on these two cell lines and found that the STIP‐silenced cells grew remarkably slower than the control shRNA‐infected cells (Fig. 2C). Colony formation assays indicated that the number of colonies was significantly reduced by 57% in A549 cells and by 40% in H460 cells as a result of STIP knockdown. Similar results were obtained in A549 cells using STIP siRNA to silence STIP expression (Fig. S1A and B). These data suggest that the down‐regulation of STIP inhibited the proliferation of NSCLC cells.

### Depletion of STIP caused cell cycle arrest at the G2/M phase by differentially regulating key cell cycle proteins

**Figure 3:**
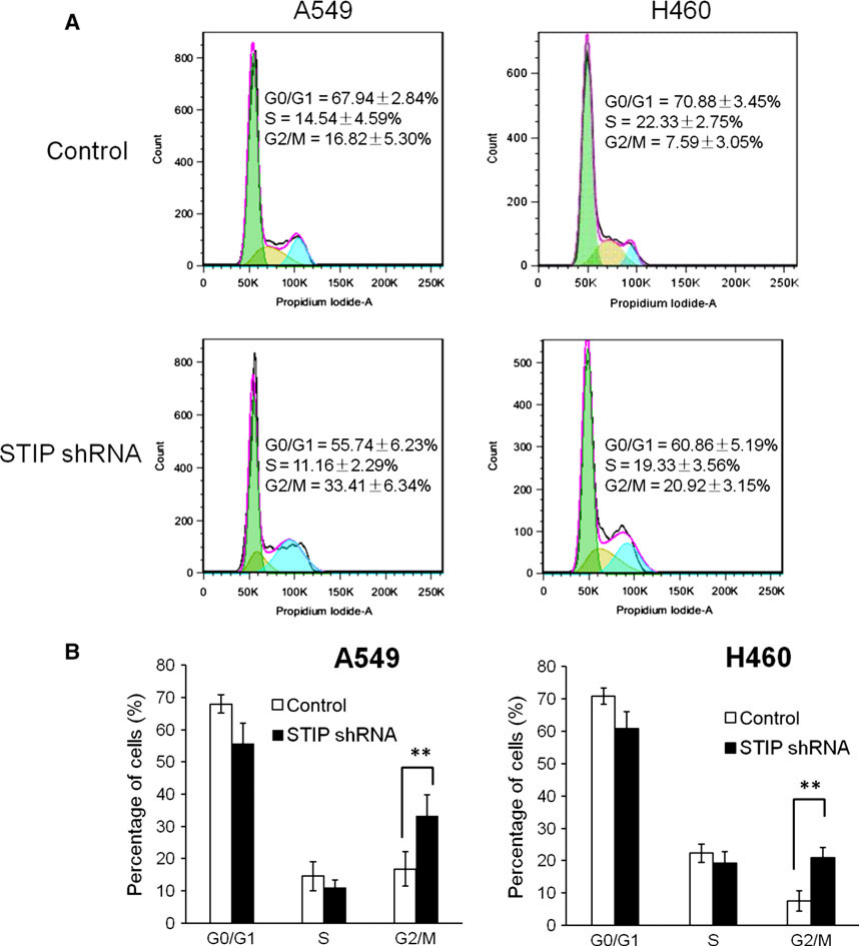
STIP knockdown induced G2/M arrest. (**A**) A549 and H460 cells were infected with STIP shRNA or control shRNA for 72 hrs and then harvested. The cells were subjected to cell cycle analysis as described in the materials and methods section. (**B**) Cell cycle distribution of A549 and H460 cells, **P < 0.01.

To explore the underlying mechanism by which STIP affects the proliferation of NSCLC cells, we first used flow cytometry to examine the effect of STIP on the cell cycle distribution of NSCLC cells. After STIP was silenced for 72 hrs in A549 and H460 cells using STIP shRNA, the cells were harvested and stained with PI for flow cytometric analysis. Compared with the control cells, the percentages of cells in the G2/M phase significantly increased from 16.82 ± 5.3% to 33.41 ± 6.34% and from 7.59 ± 3.05% to 20.92 ± 3.15% in STIP‐silenced A549 and H460 cells, respectively. These increases were accompanied by concomitant decreases in the percentages of cells in the G0/G1 or S phase (Fig. 3A and B), suggesting that STIP depletion induces cell cycle arrest at the G2/M phase.

**Figure 4:**
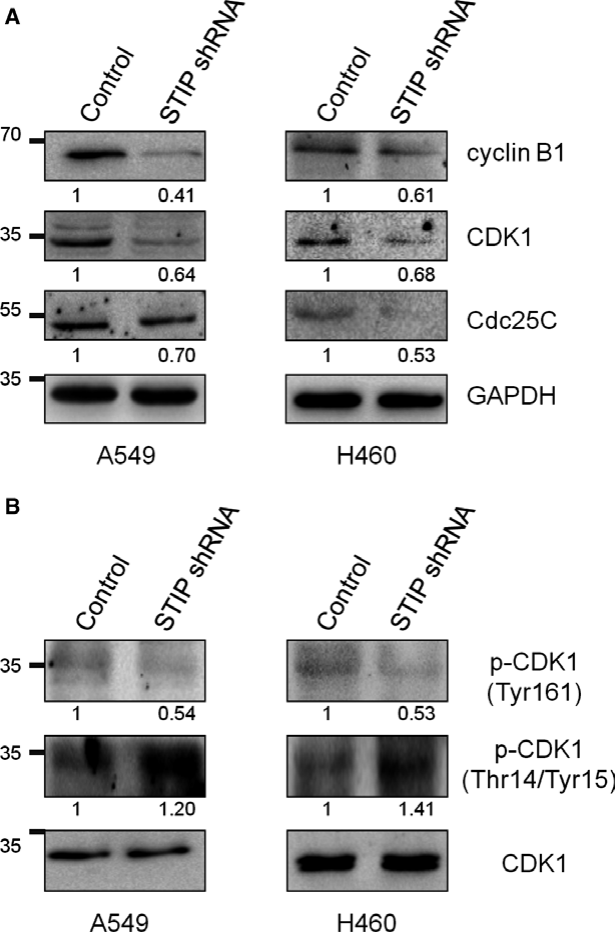
Effects of STIP on G2/M‐associated protein expression and CDK1 activity. (**A**) Total protein extracts were analysed after STIP knockdown using shRNA in A549 and H460 cells, and Western blot analyses of cyclin B1, CDK1 and Cdc25C expression were performed. GAPDH was used as an internal control. (**B**) Phosphorylation status of CDK1 in A549 and H460 cells were examined by Western blot using indicated antibodies.

To further elucidate the molecular changes associated with the observed G2/M arrest in STIP‐depleted cells, a panel of key cell cycle regulatory proteins was assessed *via* Western blot. Because the CDK1‐cyclin B1 complex plays a crucial role in the progression from G2 to M phase 16, we first tested whether STIP influences the expression of cyclin B1 and CDK1. As shown in Figure 4A, the down‐regulation of STIP resulted in a significant decrease in cyclin B1 and CDK1 protein expression in A549 and H460 cells. Because the activity of the CDK1‐cyclin B1 complex depends on the phosphorylation/dephosphorylation status of CDK1 17, the effect of STIP on the phosphorylation/dephosphorylation status of CDK1 was further investigated in A549 and H460 cells. Compared with the control cells, the phosphorylation of CDK1 at Thr‐161 was significantly reduced in the STIP‐silenced A549 and H460 cells. Alternatively, CDK1 phosphorylation at Thr‐14/Try‐15 was increased in the STIP‐silenced A549 and H460 cells (Fig. 4B). The phosphatase Cdc25C activates CDK1 by dephosphorylating CDK1 at Thr‐14/Tyr‐15 18. To verify that the increased phosphorylation of CDK1 at Thr‐14/Try‐15 was associated with Cdc25C, the expression of Cdc25C was measured in both cell lines. As expected, Cdc25C expression was significantly reduced after STIP was silenced in A549 and H460 cells using STIP shRNA (Fig. 4A). Similar results were obtained using A549 cells transfected with STIP siRNA (Fig. S2).

### Induction of apoptosis after STIP knockdown

**Figure 5:**
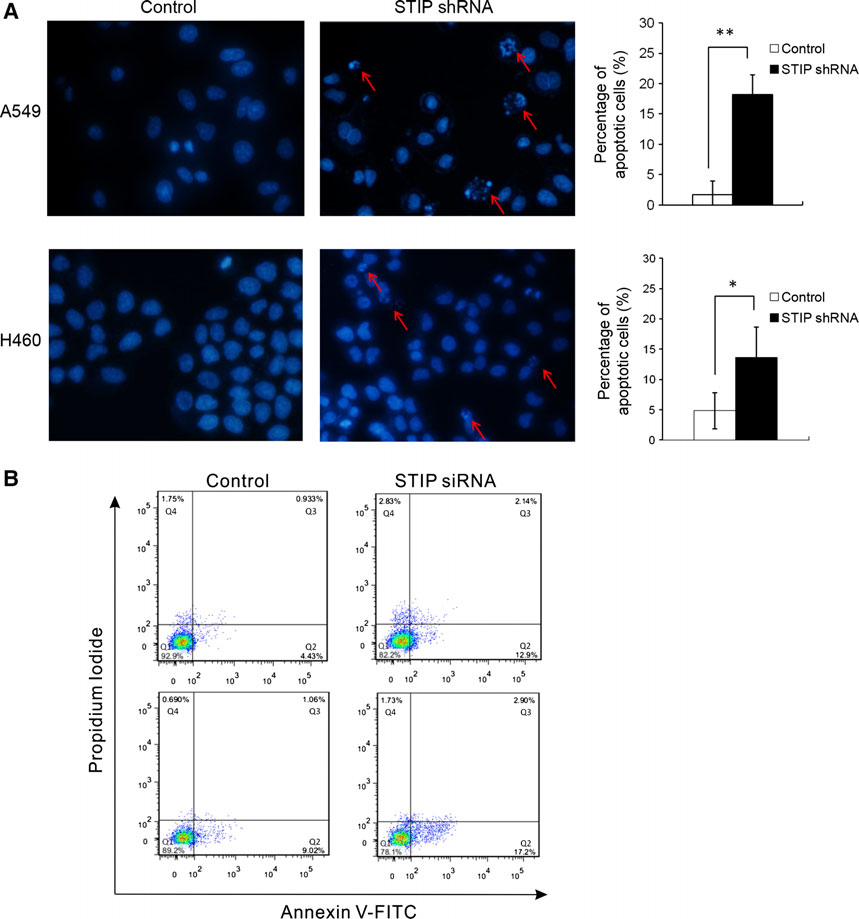
STIP inhibition caused apoptosis. (**A**) Apoptotic changes in nuclear morphology were observed *via* Hoechst 33258 staining by fluorescence microscopy. Apoptotic cells are indicated with a red arrow. The percentage of apoptotic cells is presented as the mean ± SD (*n* = 3; *P < 0.05; **P < 0.01). (**B**) A549 and H460 cells were treated with STIP siRNA for 72 hrs and then stained with Annexin V‐FITC/PI. The percentage of apoptotic cells was analysed *via* flow cytometry.

In general, cell cycle arrest is accompanied by the induction of apoptosis. To determine the role of STIP in apoptosis, nuclear morphological changes were investigated in STIP‐silenced A549 and H460 cells by staining with the fluorochrome Hoechst 33258. Based on chromatin condensation and nuclear fragmentation, a significant increase in apoptotic nuclei was observed in the STIP shRNA‐treated cells than in the control‐treated cells (Fig. 5A). Furthermore, apoptotic cells were analysed *via* flow cytometry using FITC‐conjugated Annexin V and PI double staining. Compared to the control cells, the A549 and H460 cells transfected with STIP siRNA displayed a significant increase in the percentage of early apoptotic cells (Annexin V‐positive, PI‐negative) (Fig. 5B). Thus, the knockdown of STIP triggered the apoptotic death of lung cancer cells.

### STIP knockdown induced apoptosis by activating caspases and by altering the Bax/Bcl‐2 ratio

**Figure 6:**
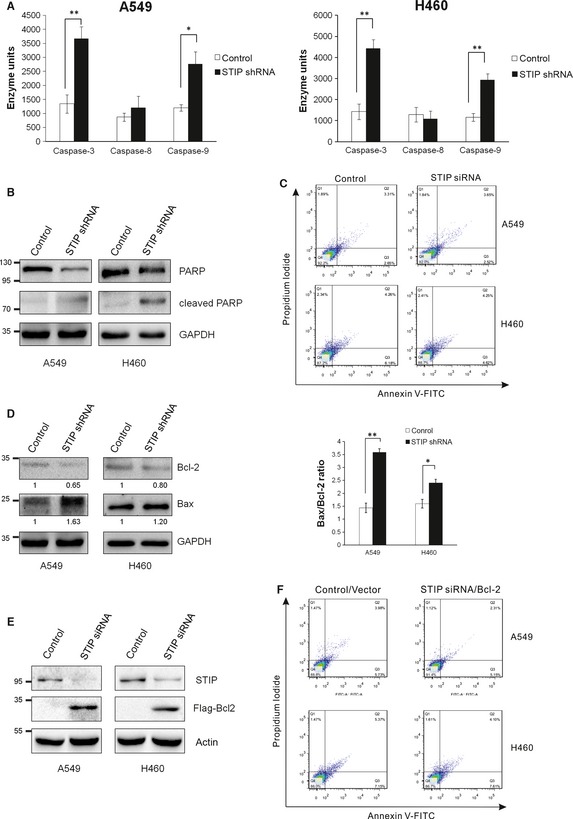
Caspases and Bcl‐2 family members were involved in STIP‐mediated apoptosis. (**A**) Cells were transfected with control shRNA or STIP shRNA for 72 hrs. Caspase‐3 and caspase‐9 activities are represented as the fold‐change relative to the control. *P < 0.05; **P < 0.01. (**B**) Cells were treated without or with STIP shRNA. Then, total cellular extracts were prepared and subjected to Western blot using an antibody against PARP. (**C**) Cells were pre‐treated with 50 μM caspase‐3 inhibitor (Ac‐DEVD‐CHO) for 3 hrs, and then transfected with STIP siRNA or control siRNA for 72 hrs. Cell were analysed for apoptosis by Annexin V‐FITC/PI two‐colour flow cytometry. (**D**) Cells were infected with STIP shRNA or control shRNA for 72 hrs. Total cellular protein extracts were prepared, and the protein expression of Bcl‐2 and Bax was analysed *via* Western blot. GAPDH was used as an internal control. The Bax/Bcl‐2 expression ratio was quantified *via* densitometry. *P < 0.05; **P < 0.01. (**E** and **F**) Cells were treated with STIP siRNA or control siRNA for 48 hrs, and then transfected with Flag‐Bcl‐2 plasmid or control plasmid for 24 hrs. (**E**) Expression of the indicated proteins were examined by Western blot. (**F**) Cell were analysed for apoptosis by Annexin V‐FITC/PI two‐colour flow cytometry.

The caspase family of proteases, which lies at the heart of the apoptotic machinery, plays a key role in the execution of apoptosis. To reveal the mechanism underlying STIP depletion‐induced apoptosis, caspase activation was evaluated in A549 and H460 cells. As shown in Figure 6A, caspase‐3 and caspase‐9 activities were significantly enhanced in STIP shRNA‐treated cells compared with negative control shRNA‐treated cells, whereas caspase‐8 activity was not affected. Furthermore, this finding was verified by measuring the cleavage of PARP, a well‐known caspase substrate. Compared with the negative control cells, the STIP‐silenced cells displayed dramatically increased levels of cleaved PARP (Fig. 6B). To demonstrate the involvement of caspase‐3 activation in the apoptotic effect, we analysed whether the caspase‐3 inhibitor Ac‐DEVD‐CHO prevented apoptosis. The results showed that caspase‐3 inhibitor Ac‐DEVD‐CHO can reverse apoptotic response in STIP‐depleted A549 and H460 cells, suggesting that STIP knockdown induced apoptosis *via* caspase‐3 activation (Fig. 6C). In addition, doxorubicin‐induced apoptosis in A549 and H460 cells were used as positive control (Fig. S3).

Bcl‐2 family proteins, including anti‐apoptotic factors, such as Bcl‐2, and pro‐apoptotic factors, such as Bax, play a pivotal role in the regulation of apoptosis 19. Accordingly, we examined the expression of Bcl‐2 and Bax in A549 and H460 cells. After STIP was silenced, the expression of Bcl‐2 was significantly decreased, whereas Bax protein expression was markedly increased, resulting in a high Bax/Bcl‐2 ratio in both cell lines (Fig. 6D). Furthermore, we found overexpressed Bcl‐2 in A549 and H460 cells could compensate for apoptosis induced by STIP knockdown (Fig. 6E and F), indicating essential roles of Bcl‐2 in STIP‐mediated apoptosis processes.

### STIP‐regulated genes are involved in multiple pathways associated with cancer

**Figure 7:**
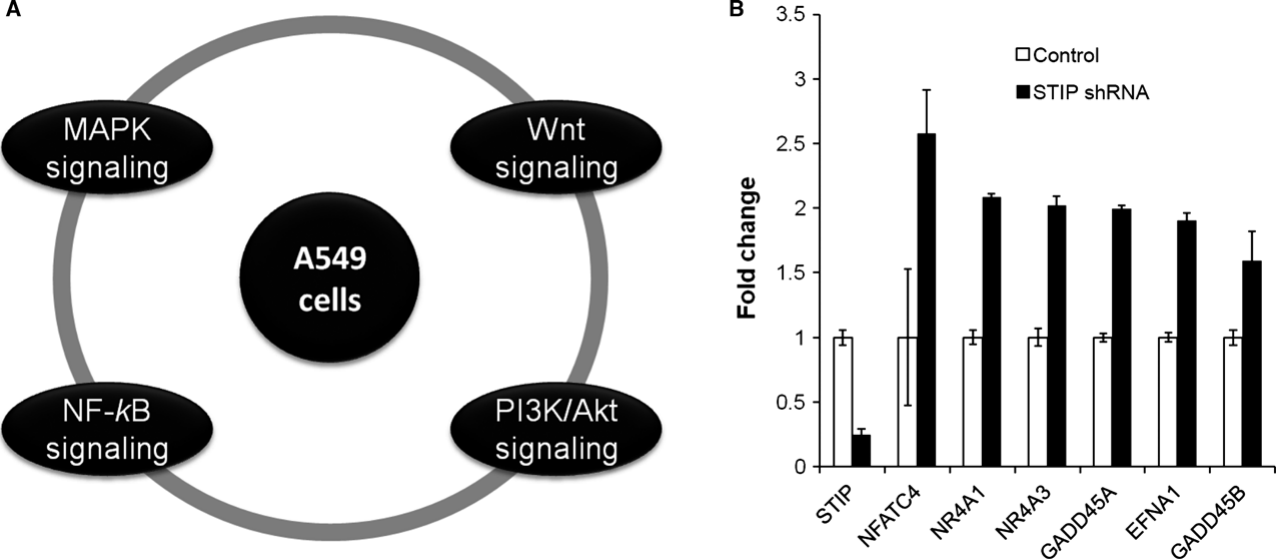
STIP influences signalling pathways and gene expression. (**A**) A549 cells were characterized using whole genome sequencing after treatment with STIP shRNA. According to the biological pathways identified by KEGG analysis, there are 4 pathways whose component genes were genetically altered in A549 cells. (**B**) Relative gene expression was assessed *via* RT‐PCR after treating A549 cells with STIP shRNA. The expression of six genes was significantly changed as evidenced by real‐time PCR.

To determine which pathways are associated with STIP‐mediated oncogenic potential in NSCLC, we investigated the changes in gene expression in A549 cells infected with STIP shRNA or control shRNA lentivirus *via* RNA sequencing. A change of twofold was used as the threshold to identify differences in gene expression. Compared with the control conditions, silencing STIP expression altered the expression of 566 genes, including the up‐regulation of 490 genes and the down‐regulation of 76 genes. The biological implications of this differential gene expression were assessed using a KEGG pathway analysis tool. The results showed that STIP depletion influenced multiple pathways associated with cancer, including the MAPK, Wnt, PI3K/AKT and NF‐κB signalling pathways (Fig. 7A). A detailed list of the genes involved in these pathways is provided in Table 2. qRT‐PCR was utilized to assess the expression of GADD45B, GADD45A, NR4A1, NR4A3, NFATC4 and EFNA1, and these results confirmed those obtained from the RNA sequencing analysis (Fig. 7B).

**Table 2:** KEGG analysis results

Regulatory pathway	Representative altered genes
MAPK signalling	GADD45A, DDIT3, NFATC4, CD14, RPS6KA2, PLA2G4A, FOS, NR4A1, GADD45B, CACNA2D1^a^
Wnt signalling	CCND2, SOX17, NFATC4, WNT11, NKD2, NKD1, FRAT1
PI3K/AKT signalling	PGF, CCND2, CASP9, CREB3L3, EFNA1, EFNA3, DDIT4, GNG7, ITGA7, CREB3L1, NGFR, NR4A3, PCK1, GNB3^a^, BRCA1^a^
NF‐κB signalling	CD40, ICAM1, CXCL2, IL8, CD14, PTGS2, TNFAIP3, GADD45B

a Indicate down‐regulated genes, and others are up‐regulated genes.

### Sustained inhibition of STIP attenuated the tumourigenic potential of lung cancer cells *in vivo*

**Figure 8:**
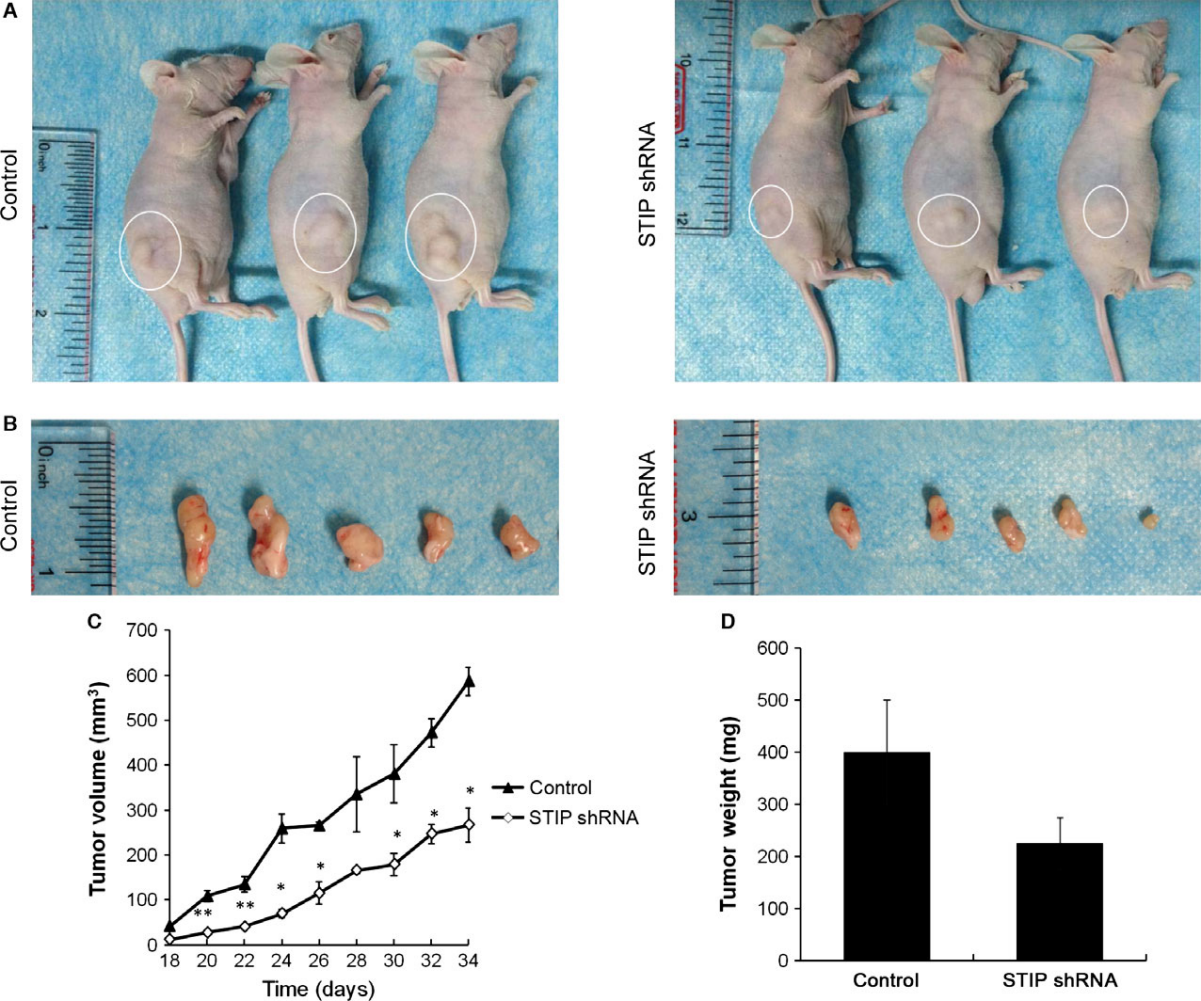
Inhibition of xenograft tumour growth *via* STIP knockdown *in vivo*. A549 cells infected with STIP or control shRNA were harvested, injected into mice, and allowed to grow for 34 days. (**A**) Representative images of human lung tumour xenografts in mice from the control and shSTIP groups. (**B**) Representative images of typical tumours from the two groups. (**C**) Tumour volumes were measured on the indicated days using the method described in the Materials and methods section. *P < 0.05; **P < 0.01. (**D**) Xenograft tumours were excised and weighed.

To further investigate the role of STIP in NSCLC *in vivo*, we established NSCLC xenografts *via* the subcutaneous injection of STIP‐silenced A549 cells or control cells into the flanks of BALB/c nude mice. After tumours appeared as palpable masses approximately 2 weeks after inoculation in the STIP knockdown or control group, tumour dimensions were measured every 2 days using callipers. The results showed that both the volume and weight of the tumours derived from STIP‐silenced cells were significantly less than those of the tumours derived from control cells (Fig. 8A–D). These data demonstrated that STIP knockdown inhibits the tumourigenicity of NSCLC *in vivo*.

## Discussion

Sip1/tuftelin‐interacting protein is a nuclear protein that is potentially associated with the spliceosome, yet its role and molecular function in cancer remain unknown. In this study, we first reported that STIP is overexpressed in clinical NSCLC tissues compared to adjacent normal lung tissues and that STIP plays a critical role in NSCLC progression by regulating cell cycle and apoptosis *via* multiple potential signalling pathways.

Cell proliferation depends on cell cycle progression, which is stimulated by the sequential activation of CDKs and their association with cyclins. Active CDK1 complexed to cyclin B1 is required for cell cycle progression from G2 to M phase. The activity of the CDK1‐cyclin B1 complex depends on the phosphorylation/dephosphorylation status of CDK1. The phosphorylation of CDK1 at Thr‐161 is required for the activation of the CDK1‐cyclin B1 kinase complex, whereas the phosphorylation of CDK1 at Thr‐14/Tyr‐15 inhibits CDK1 kinase activity 20, 21. Our studies indicated that STIP knockdown in NSCLC cells led to cell cycle arrest at the G2/M phase. The effect of STIP on the expression of G2/M‐related proteins suggested that cell cycle arrest is associated with the down‐regulation of CDK1 and cyclin B1. Furthermore, we found that STIP knockdown inhibited the activity of the CDK1‐cyclin B1 complex as reflected by a decrease in the phosphorylation of CDK1 at Thr‐161 and an increase in the phosphorylation of CDK1 at Thr‐14/Try‐15. Our results also showed that the expression of the phosphatase Cdc25C decreased after STIP was silenced. Because Cdc25C activated CDK1 *via* the dephosphorylation of CDK1 at Thr‐14/Tyr‐15, we concluded that reduced Cdc25C expression might weaken its ability to activate CDK1. Collectively, these results provide evidence that STIP knockdown in NSCLC causes cell cycle arrest at the G2/M phase by affecting the expression and activation of the CDK1‐cyclin B1 complex.

In general, cell cycle arrest is accompanied by the induction of apoptosis. We demonstrated that STIP knockdown in NSCLC cells induced apoptosis by observing nuclear morphological changes and by performing flow cytometry analysis using Annexin V and PI double staining. Cysteine proteases, which play key roles as regulators and executioners of apoptosis, include upstream (initiator) and downstream (effector) caspases. The initiator caspases, typically caspase‐8 and caspase‐9, are activated by two alternative pathways. The extrinsic pathway of apoptosis (cell death receptor‐mediated apoptosis) is responsible for the activation of caspase‐8 22, whereas the induction of the intrinsic apoptosis pathway (mitochondria‐mediated apoptosis) results in the activation of caspase‐9 23. In both pathways, an initiator caspase cleaves and activates effector caspases such as caspase‐3. Our results showed that STIP knockdown in NSCLC cells activated caspase‐9 and caspase‐3 rather than caspase‐8. The selective activation of caspase‐9 as opposed to caspase‐8 suggests that STIP knockdown induced apoptosis *via* the mitochondrial pathway 24.

Bcl‐2 family proteins are involved in the regulation of apoptosis. Pro‐apoptotic Bax mediates apoptosis by inducing cytochrome C release from mitochondria. Conversely, anti‐apoptotic Bcl‐2 blocks apoptosis by inhibiting Bax oligomerization. Studies have revealed that Bcl‐2/Bax expression ratio determines the response to an apoptotic signal 25. We found that STIP knockdown in NSCLC cells increased the expression of Bax and decreased that of Bcl‐2, resulting in a significant elevation in the Bcl‐2/Bax ratio.

Proteomic studies identified STIP as a component of the nuclear spliceosome. The spliceosome, a large complex that includes snRNPs and non‐snRNP‐associated proteins, functions in pre‐mRNA processing by excising intronic nucleic acids, thus producing mRNA that is subsequently translated into protein by ribosomes. Studies have indicated that the spliceosome can affect cell cycle progression and the induction of apoptosis, which are often impaired in cancer by altered gene expression patterns 13. To identify STIP‐mediated gene expression changes, RNA sequencing was performed on NSCLC cells. Compared with the control treatment, STIP knockdown resulted in the differential expression of 566 identified genes, including 490 up‐regulated genes and 76 down‐regulated genes. These genes are involved in multiple processes associated with cancer, including the MAPK, Wnt, PI3K/AKT and NF‐κB signalling pathways. These data provide primary clues for further research on the molecular function of STIP in NSCLC cells. However, the current data are not sufficient to support any in‐depth hypothesis. Many interesting issues remain to be resolved, including (*i*) which genes are directly or indirectly regulated by STIP, (*ii*) how STIP, as a component of the nuclear spliceosome, mediates the expression of these genes, and (*iii*) how STIP affects these signalling pathways to achieve its biological functions in NSCLC.

In summary, our results show, for the first time, that STIP was dramatically increased in NSCLC tissues and that it plays a critical role in NSCLC progression by regulating cell cycle and apoptosis *via* multiple potential signalling pathways. These findings are significant in that they provide a mechanistic framework for further exploring the use of STIP as a novel diagnostic and therapeutic target for NSCLC.

## Conflicts of interest

The authors disclose no potential conflicts of interest.

## Supporting information


**Figure S1** Silencing STIP suppressed cell proliferation.

**Figure S2** Effects of STIP knockdown on G2/M‐associated protein expression and CDK1 activity.

**Figure S3** Doxorubicin‐induced apoptosis in A549 and H460 cells.
