# Supplementary figures and images for: STIP overexpression confers oncogenic potential to human non‐small cell lung cancer cells by regulating cell cycle and apoptosis
Source: J Cell Mol Med. 2015 Sep 10;19(12):2806–17. doi: 10.1111/jcmm.12670 (PMC4687698; doi:10.1111/jcmm.12670)

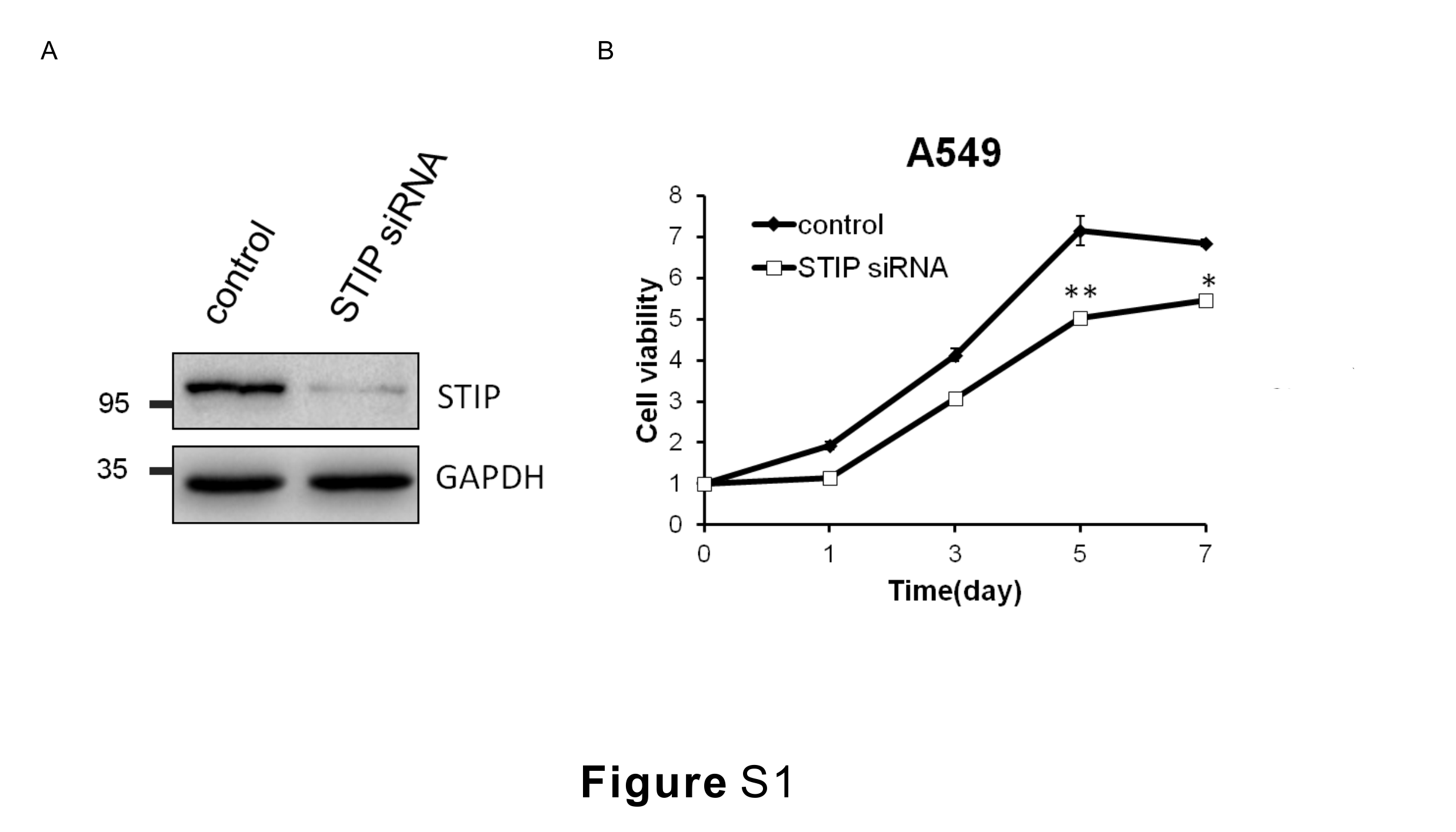

Supplement: Supplementary file 1 — Figure S1 Silencing STIP suppressed cell proliferation. [file JCMM-19-2806-s001.tif]

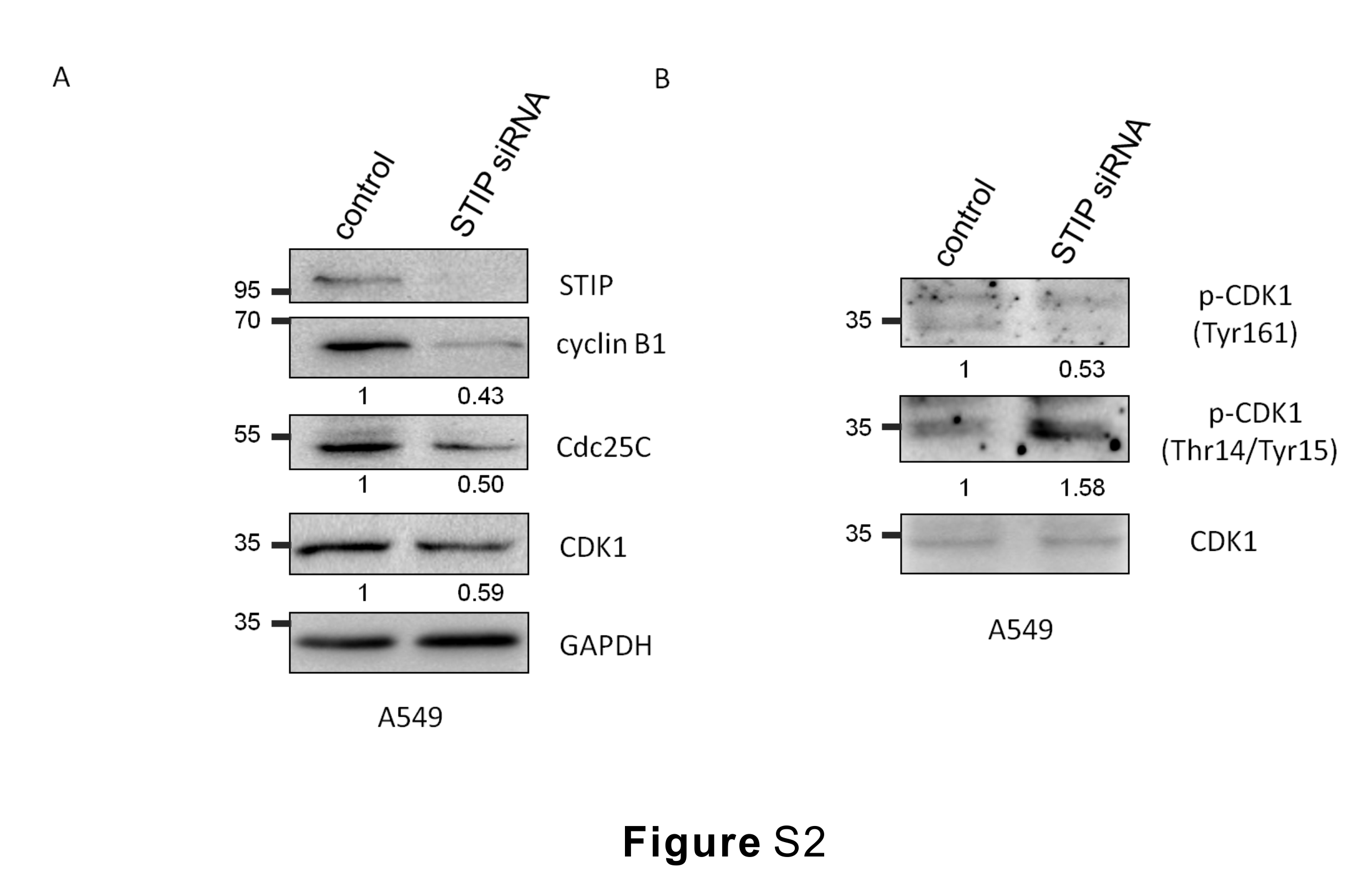

Supplement: Supplementary file 2 — Figure S2 Effects of STIP knockdown on G2/M‐associated protein expression and CDK1 activity. [file JCMM-19-2806-s002.tif]

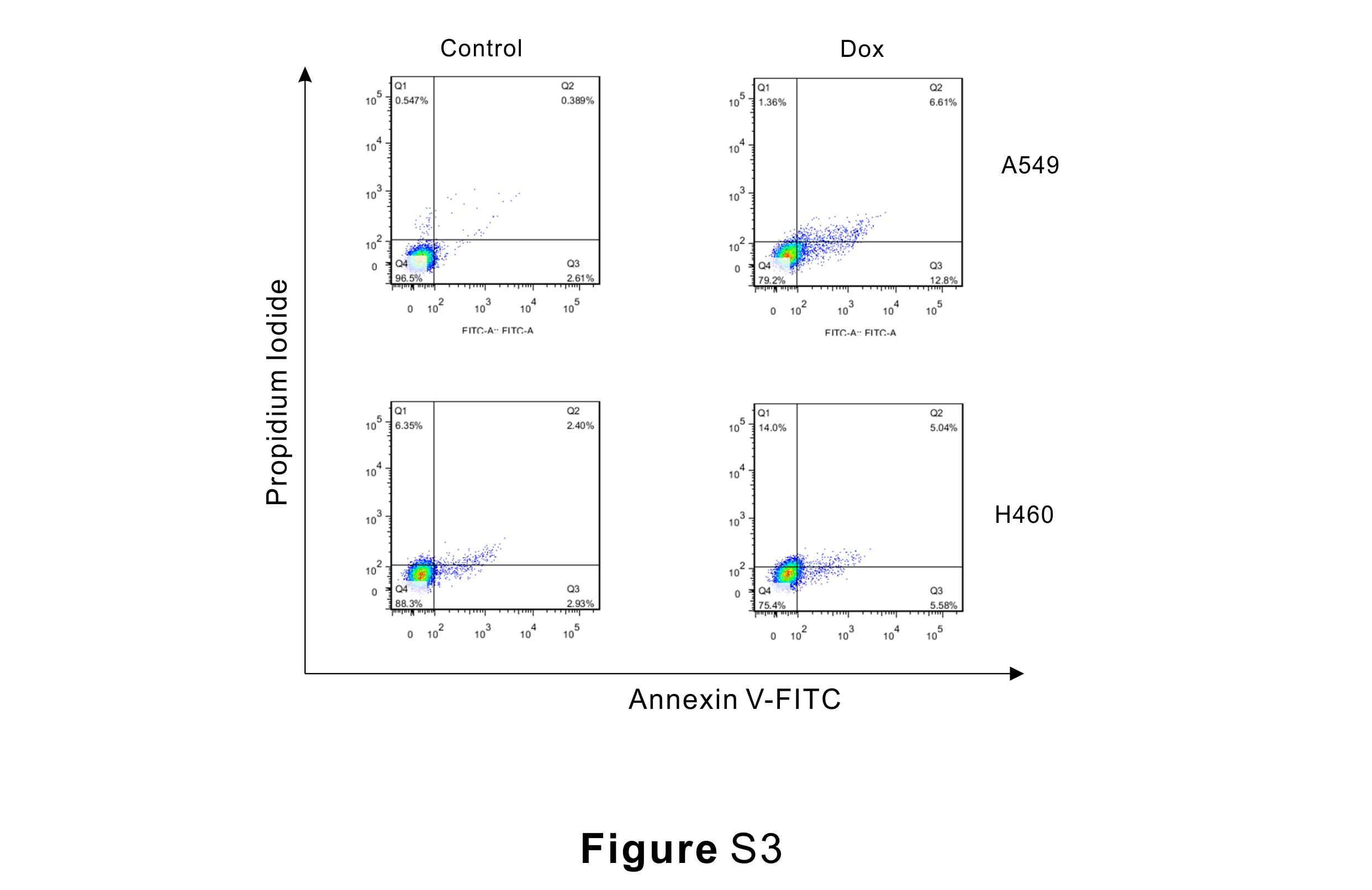

Supplement: Supplementary file 3 — Figure S3 Doxorubicin‐induced apoptosis in A549 and H460 cells. [file JCMM-19-2806-s003.tif]
