# Supplementary material for: STIP overexpression confers oncogenic potential to human non‐small cell lung cancer cells by regulating cell cycle and apoptosis
Source: J Cell Mol Med. 2015 Sep 10;19(12):2806–17. doi: 10.1111/jcmm.12670 (PMC4687698; doi:10.1111/jcmm.12670)
Supplement: Supplementary file 4 [file JCMM-19-2806-s004.doc]

**Supporting Information**

**Figure legends**

Fig. S1 Silencing STIP suppressed cell proliferation. (A) The expression of STIP protein in A549 cells were examined by western blot after transfection with STIP siRNA or control siRNA (5 nM) for 72 h. GAPDH was used as an internal control. (B) Growth curve of A549 cells after treatment with STIP siRNA or control siRNA(5 nM). The cells were plated in triplicate in six-well dishes; after plating (day 0), the cells were counted on days 1, 3, 5, and 7 using MTT assays. *P<0.05; **P<0.01.

Fig. S2 Effects of STIP knockdown on G2/M-associated protein expression and CDK1 activity. (A) Total protein extracts were prepared after STIP knockdown using siRNA (5 nM) in A549 cells. Then, cyclin B1, CDK1, and Cdc25C were analyzed via Western blot analysis. GAPDH was used as an internal control. (B) Phosphorylation status of CDK1 in A549 cells.

Fig. S3 Doxorubicin induced apoptosis in A549 and H460 cells. Cells were treated with 5μM Doxorubicin for 24h, and then stained with Annexin V-FITC/PI. The percentage of apoptotic cells was analyzed via flow cytometry.
